# Consensus statements for the biopsychosocial care of patients with epidermolysis bullosa South Africa: Part 2

**DOI:** 10.4102/hsag.v30i0.2964

**Published:** 2025-08-15

**Authors:** Antoinette V. Chateau, David Blackbeard, Carol Hlela, Martie Wege, Anne Armour, Thirona Naicker, Ncoza Dlova, Serantha Foolchand, Angela Chetty, Sarah Ainsworth, Cassidy-Mae Shaw, Reshmee Lachman, Carl-Heinz Kruse, Kavir Rajkumar, Pieter Mare, Andile Mchiza, Heidi Shanahan, Toni Roberts, Shuaib Kauchali, Colleen Aldous

**Affiliations:** 1 Department of Dermatology, Faculty of Health Sciences, Grey’s Hospital, Pietermaritzburg, South Africa; 2 Department of Dermatology, Faculty of Health Sciences, University of KwaZulu-Natal, Durban, South Africa; 3 Department of Clinical Psychology, Faculty of Health Sciences, Grey’s Hospital, Pietermaritzburg, South Africa; 4 Department of Psychiatry, Faculty of Health Sciences, University of KwaZulu-Natal, Durban, South Africa; 5 Department of Dermatology, Faculty of Health Sciences, Red Cross Children’s Hospital, Cape Town, South Africa; 6 Department of Dermatology, Faculty of Health Sciences, University of Cape Town, Cape Town, South Africa; 7 Department of Paediatrics, Faculty of Health Sciences, Grey’s Hospital, Pietermaritzburg, South Africa; 8 Department of Paediatrics, Faculty of Health Sciences, University of KwaZulu-Natal, Durban, South Africa; 9 Department of Paediatrics, Clinical Genetics, Faculty of Health Sciences, University of KwaZulu-Natal, Durban, South Africa; 10 Department of Obstetrics and Gynaecology, Maternal and Fetal Medicine, Faculty of Health Sciences, Grey’s Hospital, Pietermaritzburg, South Africa; 11 Department of Obstetrics and Gynaecology, Faculty of Health Sciences, University of KwaZulu-Natal, Durban, South Africa; 12 Department of Occupational Therapy, Faculty of Health Sciences, Grey’s Hospital, Pietermaritzburg, South Africa; 13 Department of Dietetics, Faculty of Health Sciences, Grey’s Hospital, Pietermaritzburg, South Africa; 14 Department of Ophthalmology, Faculty of Health Sciences, University of KwaZulu-Natal, Durban, South Africa; 15 Department of Dentistry and Maxillofacial Surgery, Faculty of Health Sciences, Grey’s Hospital, Pietermaritzburg, South Africa; 16 Department of Orthopaedic Surgery, Faculty of Health Sciences, Grey’s Hospital, Pietermaritzburg, South Africa; 17 Department of Podiatry, Faculty of Health Sciences, Grey’s Hospital, Pietermaritzburg, South Africa; 18 Department of Physiotherapy, Faculty of Health Sciences, Grey’s Hospital, Pietermaritzburg, South Africa; 19 DEBRA South Africa, Cape Town, South Africa; 20 Division of Community Paediatrics and Child Health, Department of Paediatrics and Child Health, School of Clinical Medicine, University of the Witwatersrand, Johannesburg, South Africa; 21 School of Clinical Medicine, Faculty of Health Sciences, University of KwaZulu-Natal, Durban, South Africa

**Keywords:** epidermolysis bullosa, multidisciplinary, cultural relevance, resource-limited, South Africa

## Abstract

**Background:**

Epidermolysis bullosa (EB) is a rare, incurable inherited mucocutaneous blistering disorder that can lead to multisystemic complications. In Africa, there are no established consensus statements or clinical guidelines for the care of patients with EB.

**Aim:**

To develop comprehensive transdisciplinary consensus statements for the care of patients with EB. This article (Part 2) presents 24 consensus statements focused on the biopsychosocial aspects of EB care. Part 1 addressed diagnostic and clinical management and resulted in 16 consensus statements.

**Setting:**

This was a multicentre, multiprovincial study involving healthcare practitioners from Eastern Cape, Free State, Gauteng, KwaZulu-Natal and Western Cape.

**Methods:**

In collaboration with patients and families, the transdisciplinary team of experts developed consensus-based statements through a modified Delphi process. This iterative process involved three consensus rounds with an 80% agreement threshold for each action point to ensure validity and reliability.

**Results:**

In total, 24 consensus statements were endorsed. These included holistic patient and family care; cultural considerations; educational inclusion; paediatric and emergency care; psychosocial care; nutritional; gynaecological and ophthalmic support; occupational therapy; physiotherapy and orthopaedic; dental and podiatric management.

**Conclusion:**

Comprehensive transdisciplinary care is essential for addressing the holistic needs of patients with EB and their families, particularly in resource-limited and culturally diverse settings.

**Contribution:**

This is the first set of consensus statements for the care of EB in South Africa and the broader African continent, offering a culturally sensitive, patient-centred framework for multidisciplinary care.

## Introduction

Epidermolysis bullosa (EB) is a rare and debilitating genetic skin disorder characterised by extreme blistering of the skin with four main types: EB simplex, junctional EB, dystrophic EB and Kindler syndrome. The severity of EB can range from mild blistering to life-threatening complications (Fine & Mellerio 2009a, 2009b), all of which can significantly impact the quality of life of patients and their families (Martin et al. 2019).

While there is no cure for EB, its management focuses on wound prevention, intensive skin care, pain control, nutritional optimisation and proactive monitoring, to prevent further complications.

Given the complexity of EB, a patient-centred care requires a transdisciplinary approach that is essential. This approach involves collaboration between healthcare professionals and allied practitioners and the patient’s family, ensuring that the physical and psychological needs of the patient are addressed while respecting their cultural beliefs. In this model, patients are actively involved in decision-making processes, empowering them and their families to contribute to their care plan (Bardes 2012; Boyd & Lucas 2014).

The psychosocial impact of EB is profound, often extending beyond physical manifestations of the disease. Patients with EB may face challenges in socialising, navigating school and work environments, enduring bullying and forming lasting relationships. Psychological support, therefore, is essential not only for the patient but also for their family unit, including parents and siblings, to help them cope with these challenges.

For children with EB, schooling can be both physically and emotionally difficult. Many educational environments are not equipped to accommodate students with special needs, making it crucial for educators and school administrators to be aware of the needs of learners with special needs such as EB. A supportive educational framework is vital to ensure these students can learn and grow in an inclusive environment.

Paediatricians play a vital role in ensuring that children with EB receive adequate nutrition, which is crucial for wound healing. They work in close consultation with dieticians, monitoring for complications and facilitating referrals to other healthcare providers, such as orthopaedic surgeons, ophthalmologists, physiotherapists, occupational therapists, dentists and podiatrists. Moreover, sexual and reproductive health is vital, empowering patients to make informed decisions about their health and future.

In South Africa (SA), many patients seek the help of a traditional health practitioner (THP) because of factors such as accessibility, confidentiality, trustworthiness and affordability (Mutola, Pemunta & Ngo 2021). However, there is often scepticism among allopathic healthcare practitioners (HCPs) about the safety and efficacy of traditional treatments (Mokgobi 2013) because of a lack of standardisation regarding dosage, frequency, duration and side effect profile of treatment (Flint & Payne 2013; Mutola et al. 2021). Despite these concerns, THPs and allopathic practitioners can help provide comprehensive care that is sensitive to cultural beliefs (Chateau et al. 2023c). Thus, healthcare professionals, both allopathic and traditional, must work collaboratively as a transdisciplinary team, ensuring that all aspects of patients’ physical, emotional and cultural beliefs are met. The holistic approach is essential for improving the quality of life of patients with EB and their families in resource-limited environments.

## Research methods and design

A task team comprising dermatologists and paediatricians convened to define the aims and methodological framework for the development of consensus statements addressing the care of patients with EB in SA. It was agreed that the final output will be presented in two documents: Part 1 (Chateau et al. 2025) and Part 2.

Part 1, focused on diagnostics and therapeutics, involved 19 specialists, including dermatologists, paediatricians, palliative care and pain specialists, a geneticist and a genetic counsellor.

Part 2, which forms the basis of this manuscript, addressed the biopsychosocial aspects of EB care.

A transdisciplinary team of 20 participants was invited to contribute, including patients with EB, dermatologists, paediatricians. emergency care specialists, clinical psychologists, an orthopaedic surgeon, an ophthalmologist, a dietician, an occupational therapist, a physiotherapist, a gynaecologist, a social worker, a dentist and a podiatrist.

The team met virtually via Zoom on 13 December 2022. Each discipline was responsible for drafting a set of topic-specific questions, which were circulated using a Google Form, to generate an initial round of consensus. In line with a patient-centred care approach, the proposed topics were subsequently reviewed by patients with EB through Dystrophic Epidermolysis Bullosa Research Association (DEBRA) SA, to ensure relevance and practicality. Based on patient feedback, additional specialist sections, such as ophthalmology and sexual health, were incorporated. Patients participated in all subsequent consensus rounds.

A comprehensive literature review was undertaken for both Part 1 and Part 2 of the referencing EB-CLINET clinical guidelines (https://www.eb-clinet.org/clinical-guidelines/), DEBRA international resources (https://www.debra-international.org/) and databases such as PubMed, EBSCOhost, Google Scholar, ClinicalKey and Wiley Online Library.

A modified Delphi technique was used to achieve consensus, following Accurate Consensus Reporting Document (ACCORD) methodology described by Gattrell et al. (2024). Three rounds of discussion were conducted with a consensus threshold of 80% set for each action point to ensure validity and reliability of the final recommendations. All 24 consensus statements developed for Part 2 were accepted and are included in this document, alongside 16 statements from Part 1.

### Ethical considerations

An application for full ethical approval was made to the Biomedical Research Ethics Committee of University of KwaZulu-Natal and consent was received on 07 May 2022 (reference no: BREC/00003768/2022).

## Results

The consensus process, guided by the modified Delphi technique, resulted in the development of 24 consensus statements addressing the biopsychosocial and transdisciplinary care of patients with EB in SA. The statements are intended to complement the 16 clinical care and management statements developed in Part 1 of this project.

As summarised in Table 1, the 24 statements are organised into 7 overarching categories, which represent an initial framework to differentiate the multifaceted and sometimes overlapping aspects of EB care in resource-limited settings. These categories emerged organically through iterative rounds of consensus-building and reflected the holistic, patient-centred care agenda endorsed by the task team and participating patients.

**TABLE 1:** Overview of the consensus statements.

Consensus statements	Total (%)	References
**A. Holistic care of the patient with epidermolysis bullosa (EB)**		
*Consensus statement 1*: Patients’ perspectives are pivotal in developing consensus statements and guidelines, supporting and advocating for patients, and educating healthcare practitioners (HCPs), patients and the community	**88**	Patients’ statements Boyd and Lucas (2014), Bardes (2012)
*Consensus statement 2*: Psychosocial support for patients and families with EB is vital for holistic well-being	**100**	Wu, Sun and Lee (2020), Kearney, Donohoe and Mcauliffe (2020) Chateau et al. (2024)
*Consensus statement 3*: It is crucial to understand and support parents who care for children with rare, incurable diseases such as EB	**100**	Chateau et al. (2023a)
*Consensus statement 4*: It is vital to educate HCPs to diagnose and manage EB. It is essential to support HCPs caring for EB patients emotionally.	**88**	Chateau et al. (2023b, 2024), Martin et al. (2019), Kearney et al. (2020), Ireland, Pelentsov and Kopecki (2021)
*Consensus statement 5*: Recognising cultural perspectives and collaboration with traditional healthcare practitioners is imperative	**100**	Zuma et al. (2016)
**B. Schooling and education for the patient with EB**		
*Consensus statement 6*: Supporting students with EB in school: A guide for schools and educators.	**94**	Van Scheppingen et al. (2008), Chernyshov et al. (2024); DEBRA international
**C. Management of the paediatric patient with EB**		
*Consensus statement 7*: A paediatrician’s integrated approach, collaboration with multidisciplinary teams and ensuring a transition to adult care.	**94**	Han et al. (2023), Wasserman et al. (2023), Fine and Mellerio (2009b), Liy-Wong et al. (2023), Department of Health (2023), Haynes (2010), Fine and Mellerio (2009b)
*Consensus statement 8*: Awareness of emergency management of EB	**100**	Mellerio et al. (2020), Goldschneider et al. (2014), Fine and Mellerio (2009a), Hachem et al. (2014), Arifi et al. 2013, Department of Health (2023), Liy-Wong et al. (2023), El Hachem et al. (2014)
**D. Nutrition for EB patients**		
*Consensus statement 9*: Optimising nutritional status for growth, development and wound healing	**94**	Manjunath et al. (2021), Sal era et al. (2020), Haynes (2008, 2010), El Hachem et al. (2014)
*Consensus statement 10*: Enteral feeding in patients with severe EB	**88**	Salera et al. (2020), El Hachem et al. (2014), Manjunath et al. (2021)
*Consensus statement 11*: Preventing and managing constipation in patients with EB	**94**	Haynes (2010), Hubbard, Mayre-Chilton and Jones (2020)
*Consensus statement 12*: Clinical and investigative monitoring of macro and micronutrients in EB patients	**88**	Sklar and Haynes (2014), Salera et al. (2020), Martinez and Mellerio (2010), Liy-Wong et al. (2023), Pope et al. (2012)
**E. Obstetric care of the pregnant patient with EB**		
*Consensus statement 13*: Diligent antenatal care of women with EB is essential to prevent trauma and complications	**94**	Araújo et al. (2017), Baloch et al. (2008), Ressler-Maerlender, Krishna and Robison (2005), Pillay (2006)m Intong et al. (2017), Greenblatt et al. (2022)
*Consensus statement 14*: Vigilant intrapartum care is important to reduce the risk of injury to the expectant mother Practical steps to follow to avoid injury Pain management strategies depending on the mode of delivery: Entonox, relaxation techniquesg and epidural and general anaesthesia The choice of delivery: vaginal and caesarean section	**100**	Shah, Kumaraswami and Mushi (2019), Greenblatt et al. (2022), Goldschneider et al. (2014), Intong et al. (2017)
*Consensus statement 15*: Postpartum care and discharge planning.	**100**	Intong et al. (2017), Greenblatt et al. (2022), Shah et al. (2019)
**F. Sexual and reproductive health and EB**		
*Consensus statement 16*: Health education on sexual health and monitoring for complications	**100**	King et al. (2021)
*Consensus statement 17*: Advice for teenagers on menstruation and use of sanitary products	**94**	Patients’ experiences
*Consensus statement 18*: Medical circumcision is not contraindicated in males with EB	**82**	Jesus et al. (2014)
**G. Other multidisciplinary teams involved in the management of patients with EB: eye care, occupation therapy, physiotherapy, orthopaedic management, oral health and footcare**		
*Consensus statement 19*: Vigilant monitoring for eye symptoms in EB is essential	**88**	Bachir et al. (2022), Fine et al. (2004a), Figueira, Murrell and Coroneo (2010)
*Consensus statement 20*: The role of occupational therapy in supporting patients with EB	**100**	Chan et al. (2019), Eismann, Lucky and Cornwall (2014)
*Consensus statement 21*: Preventive strategies and management of orthopaedic complications in Recessive Dystrophic Epidermolysis Bullosa (RDEB)	**94**	Box et al. (2022), Eismann et al. (2014), Denyer et al. (2017), El Hachem et al. (2014), Bernardis and Box (2010), Sternick et al. (2016)
*Consensus statement 22*: Physiotherapy for patients with EB: enhancing mobility, preventing complications and promoting inclusivity	**94**	El Hachem et al. (2014), Weisman et al. (2021), Mullett and Atherton (1990), Box et al. (2022)
*Consensus statement 23*: Good oral health, trauma prevention and monitoring for oral complications in EB	**100**	Krämer et al. (2020), Has et al. (2021), Feijoo et al. (2011)
*Consensus statement 24*: Footcare in EB – prevention and management	**100**	Khan et al. (2020), DEBRA International

Note: Please see full reference list of this article: Chateau, A.V., Blackbeard, D., Hlela, C., Wege, M., Armour, L.A., Naicker, T. et al., 2025, ‘Consensus statements for the biopsychosocial care of patients with epidermolysis bullosa South Africa: Part 2’, *Health SA Gesondheid* 30(0), a2964. https://doi.org/10.4102/hsag.v30i0.2964 for more information.

The seven categories are as follows: *Holistic care and family support:* emphasising a person- and family-centred model of care that addresses the emotional, social and practical needs of patients and their caregivers. *The psychological and cultural considerations:* highlighting the importance of integrating psychological support, mental health care and cultural sensitivity into EB management. *Education and Schooling:* addressing the rights of children with EB to inclusive education and support systems needed to facilitate learning in various settings. *Nutritional support*: recognising the role of diet and feeding support in maintaining health and promoting wound healing in patients with EB; *Paediatric care:* providing guidance on age-appropriate, developmentally sensitive management of children with EB, particularly in early stages; *Obstetric and reproductive health*: offering recommendations for obstetric care of pregnant patients with EB and addressing their reproductive and sexual health; *Specialist subspeciality care:* outlining the roles of diverse specialists, including orthopaedics, ophthalmology, a physiotherapy, dentistry and others, to ensure coordinated transdisciplinary care.

Each category reflects the complexity and breadth of EB care, and together they offer a comprehensiveness that integrates clinical expertise with lived experience. The consensus statements were shaped by inputs from patients, healthcare providers and allied professionals, ensuring their relevance, feasibility and cultural appropriateness within the South African context.

### Holistic care of the patient with epidermolysis bullosa

#### Consensus statement 1: Patient perspectives are pivotal in developing consensus statements and guidelines, supporting and advocating for patients, and educating healthcare practitioners, patients and the community

The patient’s view and input are fundamental in developing consensus statements and guidelines. They live with the condition daily and are in the best position to advise on their needs, concerns, symptomatology, complications, management needs and their vision going forward. Support groups are pivotal in supporting patients and families, are role players in policy-making, advocate for patients and educate society.

Support to the patients and families

- DEBRA SA is a local support group that aims to assist those living with EB in SA. It has many members across the county. They offer invaluable emotional support by fostering a sense of community among patients, caregivers and families. By connecting people with shared experiences, DEBRA SA creates a supportive network where individuals can share knowledge, experiences and coping strategies. They work closely with doctors and dermatologists to offer new patients and their families the best possible care.
- By combining their expertise, resources and dedication, they have established a comprehensive support network that addresses EB patients’ unique challenges in SA. Together, they provide essential medical assistance, access to online specialised care portals and vital information about EB to patients and their families.

**Education of healthcare practitioners, patients and families**

- DEBRA International has created clinical practice guidelines, of which DEBRA SA has played a vital role in the development process. These guidelines offer EB patients insights into managing every sphere of life affected by EB. To support developing nations, they have also published an ‘infographic’ version, which contains primarily images and minimal text to be more inclusive with their support.

**Raising awareness about epidermolysis bullosa**

- DEBRA SA is pivotal in raising awareness about EB within the medical community and the general public. Their awareness campaigns help educate people about the challenges faced by EB patients, fostering understanding and empathy.

**Advocating for patients**

- DEBRA SA also advocates for improved healthcare services and research initiatives, working closely with medical professionals and policymakers to enhance the standard of care for EB.

**The vision of Dystrophic Epidermolysis Bullosa Research Association South Africa**

- Beyond emotional support, awareness campaigns, educational programmes and advocacy efforts, in the future, DEBRA SA aims to have the capacity to organise various funding opportunities to provide financial assistance to patients. This financial support would aid in covering medical expenses, specialised treatments and essential medical supplies, relieving some of the burdens EB patients and their families face.

#### Consensus statement 2: Psychosocial support for patients and families with epidermolysis bullosa is essential for holistic well-being

Psychological support for EB patients is essential for the holistic well-being of patients with EB and their families. Enhancing psychological support begins with improving patient–physician interactions, fostering collaborative care and empowering patients as active participants in managing their condition. This balance between self-efficacy and specialist support helps reduce psychological pain and fosters confidence and motivation (Martin et al. 2019). Healthcare professionals must ensure that they are educated and informed with a high level of knowledge to provide expert support and guidance. This will minimise the psychological strain placed on patients to be their own experts (Chateau et al. 2023b, 2024).

The key aspects of psychological support include:

- **Early and dynamic psychological care:**
  ■ Screening, monitoring and managing psychological needs should begin at diagnosis and adapt to the patient’s developmental stage and life transitions (Chateau et al. 2023b; Martin et al. 2019).
  ■ Early psychological support improves coping strategies, prevents mental health deterioration and minimises psychological disorders (Ireland et al. 2021).

- **Tailored and holistic care:** (Chateau et al. 2024; Ireland et al. 2021)
  ■ Support must consider the unique social, physical, practical and psychological needs of patients.
  ■ Factors such as disease severity, access to medical care and family dynamics profoundly affect mental health.

- **Family support:**
  ■ Parents often experience guilt, helplessness and isolation, especially when caring for a child with special needs (Ireland et al. 2021; Kearney et al. 2020).
  ■ Educating parents about coping strategies, sharing caregiving responsibilities and strengthening family cohesion can improve psychological outcomes for all members (Chateau et al. 2023b; Kearney et al. 2020; Martin et al. 2019).
  ■ Difficulties accessing basic medical care and necessities place a substantial psychological strain on families (Kearney et al. 2020).
  ■ Emotional strain on parents because of role-switching between caregiver and parent must be acknowledged and addressed (Martin et al. 2019).

- **Professional collaboration:**
  ■ Multidisciplinary collaboration is crucial for addressing patients’ needs holistically and supporting healthcare providers in managing the emotional demands of care, thereby preventing burnout (Martin et al. 2019).
  ■ Referrals for psychological and psychiatric care should be made when anxiety, depression or self-esteem issues arise.

- **Financial and social support:**
  ■ Social workers can assist families in accessing financial aid through government grants (e.g., care dependency, caregiver and disability grants) via South African Social Security Agency (SASSA) and non-governmental organisations (NGOs).
  ■ Home visits and needs assessments help ensure families receive resources necessary to alleviate financial strain.

Impact on psychological well-being: holistic and collaborative care fosters improved psychological outcomes, addressing key challenges such as anxiety, depression and self-esteem issues. By supporting both patients and families, healthcare providers can enhance the quality of those living with EB while addressing the broader emotional, financial and social challenges they face.

#### Consensus statement 3: It is crucial to understand and support parents who care for children with rare, incurable diseases such as epidermolysis bullosa

Caring for a child with a rare and painful skin condition such as EB is emotionally and physically overwhelming for many parents. They may experience a wide range of emotions, including joy, guilt, fear, sadness and helplessness (Wu et al. 2020). Guilt often stems from feeling responsible for their child’s pain during dressing changes or passing on the genetic condition (Kearney et al. 2020).

Delay in diagnosis and inadequate knowledge among HCPs can exacerbate the condition, leading to disease progression and added stress for parents. Some parents report feeling judged or blamed by HCPs for their child’s injury, mistaking bandaging for harm or neglect. The high care demands also place significant strain on families. (Chateau et al. 2024) Caring for a child with high needs may place a significant financial burden on the family (Wu et al. 2020):

- Parent support needs include financial assistance and referrals to social services for care dependency grants.
- Active involvement in decisions regarding their child’s care.
- Education and managing EB, including blister prevention and complication management.
- Access to psychological support for parents and caregivers.
- Holistic family care that respects cultural beliefs and practices.
- Acknowledgement and consideration of the parent’s needs, concerns and suggestions.

Proving empathetic and comprehensive support can alleviate the burden on families and improve their quality of life.

#### Consensus statement 4: It is vital to educate healthcare practitioners to diagnose and manage epidermolysis bullosa. It is essential to support healthcare practitioners caring for patients with epidermolysis bullosa

Epidermolysis bullosa is a rare disease, and many HCPs lack experience in its diagnosis and management. The gap can lead to delays in care, increased complications in care and worsened outcomes for patients, particularly in severe types with high mortality rates. In addition, the emotional toll of caring for these patients can negatively affect HCPs increasing their risk of burnout (Chateau et al. 2023a).

Key recommendations:

- Provide education and training for HCPs on diagnosing and management of EB, including wound prevention and care, through consensus guidelines, workshops and continuing medical education seminars.
- Offer support for HCPs, including debriefing sessions and referral to employee-assisted programmes or clinical psychologists to address emotional strain and burnout.

Empowering and supporting HCPs is essential to improve patient outcomes and ensure sustainable compassionate care.

#### Consensus statement 5: Recognising cultural perspectives and collaboration with traditional healthcare practitioners is imperative

Traditional healthcare practitioners play a significant role in healthcare, with up to 70% of the African population consulting THPs before seeking care from allopathic healthcare providers (Zuma et al. 2016).

**Key recommendations**

- Acknowledge the role of THPs: recognise the valuable contribution THPs make to the healthcare system.
- Foster collaboration: encourage collaboration between the HCPs and THPs to enhance holistic care for patients.
- Provide education on EB: Educate THPs about EB, emphasising the risks of practices such as applying herbs on the skin, performing scarifications or using enemas, which may exacerbate the condition and lead to complications.

A culturally sensitive and collaborative approach ensures that patients receive comprehensive care while respecting their cultural beliefs and practices.

### Schooling and education for the patient with epidermolysis bullosa

#### Consensus statement 6: Supporting students with epidermolysis bullosa in school: A guide for schools and educators

**Challenges faced by students with epidermolysis bullosa:**

- Many mainstream schools lack the necessary adaptations for high-need students, and specialised schools are often unaffordable, especially in SA.
- Physical discomfort and pain from navigating the school environment.
- Increased absenteeism because of pain, infections and other complications.
- Social stigma, including bullying, teasing and accusations of being contagious, leading to long-term psychological and social consequences such as depression, anxiety and suicidal ideation (Chernyshov et al. 2024; Van Scheppingen et al. 2008).

The teachers and the school nurse need to be educated as to the potential complications and the necessary adaptations that may need to be made (DEBRA of America 2005 international).

**Recommendations for schools**

**Education for staff**

- Teachers: understand the challenges faced by students with EB, including physical, academic and emotional needs.
- School nurses: training in wound care, lancing blisters, applying dressings, administering medication, including managing minor complications.

**Classroom adaptations**

- Provide cushions to prevent blister formation from prolonged sitting.
- Allow extra time for writing tasks or navigating between classrooms.
- Reduce weight for school bags or offer alternatives such as trolleys.
- Permit longer meal times because of difficulties eating.

**Physical education and activities**

- Modify activities to reduce risk while encouraging inclusion.
- Address concerns about body exposure and ensure a safe environment for participation.

**School trips**

- Provide accommodation such as wheelchairs for long distances.
- Educate staff on managing potential complications during outings.

**Uniform modifications**

- Allow soft, comfortable clothing and footwear.
- Permit the use of long sleeves or pants to cover bandages.

**Psychological support**

- Offer counselling for students to cope with challenges and self-esteem issues.
- Address bullying proactively and create an inclusive school environment.

**Support during transitions**

- Recognise the increased workload and emotional demands of moving to secondary school.
- Provide additional academic and emotional support during this period.
- *Holistic school support*: by educating staff, adapting the environment and fostering an inclusive culture to fostering inclusivity, schools can help EB patients feel safe, supported and empowered to succeed academically and socially.

### Management of the paediatric patient with epidermolysis bullosa

#### Consensus statement 7: The role of the paediatrician in identifying and managing epidermolysis bullosa

Paediatricians’ integrated approach and collaboration with dermatologists should play a leading role in preventing, identifying and managing complications and facilitating care with other HCPs to improve the quality of life of patients.

Key responsibilities of the paediatrician include:

- providing adequate pain management.
- assisting families in accessing social support such as care dependency grant for severe EB cases.
- discussion with the palliative care team to ensure early involvement and development of care plans (when applicable), engaging palliative care teams early to develop care plans as needed (see section on palliative care).
- offering ongoing follow-up to monitor and manage complications.
- supporting the transition to adolescent and adult care.

**Transition of care:** Transition of care from adolescence to adulthood must be planned with the involvement of the adult physicians. Open communication bridges knowledge gaps and ensures a seamless shift in care. Combined clinics during adolescence featuring both paediatric and adult care teams can improve outcomes (Han et al. 2023). The goal for managing EB patients should be to enhance the quality of life.

**Managing complications:** Paediatricians are instrumental in identifying and addressing EB complications, including:

- ***Delayed Puberty*:** chronic inflammation and malnutrition can delay puberty and reduce bone mineralisation, increasing osteoporosis risk. Regular pubertal screening and bone health monitoring are essential (Fine & Mellerio 2009b; Wasserman et al. 2023).
- ***Anaemia*:** anaemia in EB is multifactorial, caused by chronic inflammation, iron deficiency, blood loss and malabsorption (Fine & Mellerio 2009b). Proper management improves wound healing, growth and quality of life. Severe iron deficiency may exacerbate gastrointestinal issues, perpetuating anaemia. Screening should occur at diagnosis in severe cases, at 1 year in moderate cases, and when symptomatic in EB simplex. The target haemoglobin level is > 10 g/dL (Liy-Wong et al. 2023).
- ***Nutrition*:** Children with EB are highly susceptible to nutritional compromise because of factors such as inflammation, infection, high energy needs for wound healing, accelerated skin turnover and gastrointestinal complications affecting intake and absorption. Early involvement of a dietician is essential (Haynes 2010).
  **Key considerations include**
  ■ Regular monitoring of weight gain using age-appropriate growth charts.
  ■ Prompt dietician intervention if growth concerns arise.
  ■ Assessment of serum albumin, iron studies and electrolytes in severe cases.
- ***Sepsis*:** Skin infection is common in all subtypes of EB, placing patients at a substantial risk for sepsis, a leading cause of mortality (Fine & Mellerio 2009b).
  **Management strategies include**
  ■ Use of broad-spectrum antibiotics to address infections.
  ■ Adjusting empiric antibiotic treatment based on institutional microbial profiles, bacterial culture results and antibiograms, particularly in cases of nosocomial infections.

Timely and coordinated management of nutrition and sepsis is crucial in optimising outcomes for children with EB. (*See antibiotic use in the next section, Table 3: Emergency management in EB*.)

**FIGURE 1:**
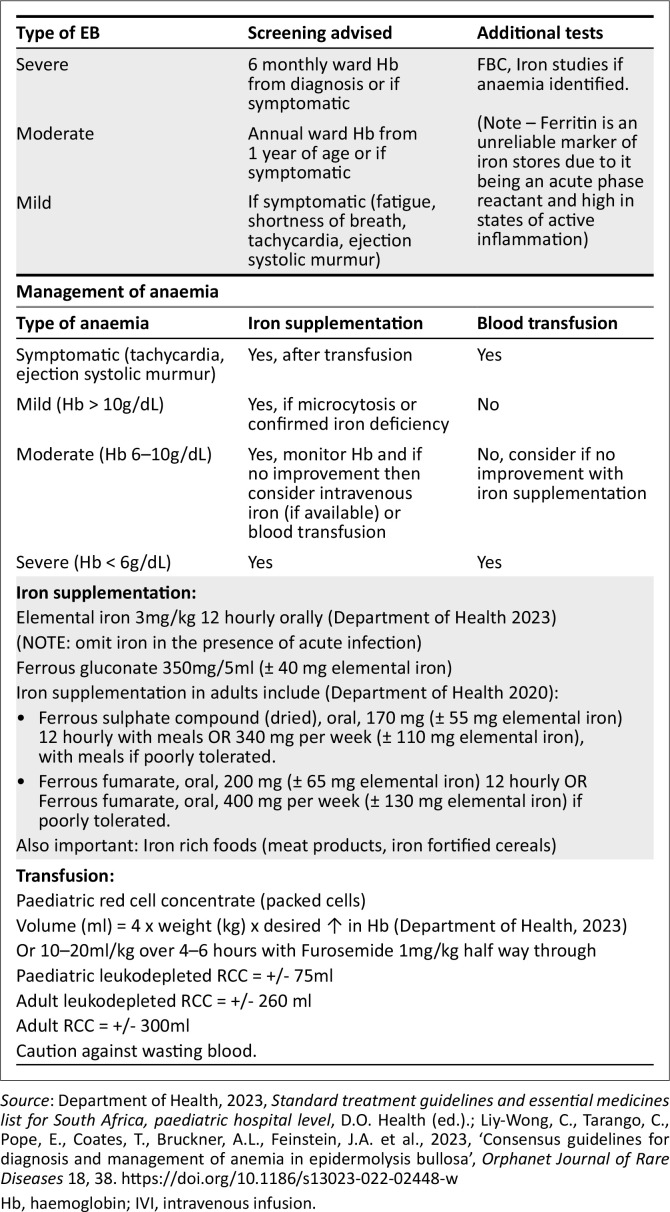
Approach to anaemia in children with epidermolysis bullosa.

Figure 1 outlines an approach to the screening, investigations and management of anaemia in children with EB.

#### Consensus statement 8: Awareness of emergency management of epidermolysis bullosa

Healthcare practitioners should be aware of the emergency management of EB, as its complications can be life-threatening and may require urgent intervention. Mellerio et al. (2020) have outlined guidelines for emergency care specific to EB patients. Because these patients may rapidly deteriorate, they might see care at local clinics or district hospitals where subspecialty expertise may not be readily available.

**Nature of emergencies in epidermolysis bullosa:** Patients with EB may present with life-threatening emergencies stemming from skin and mucosal blistering or secondary complications because of extracutaneous manifestations. While EB has no cure, emergency management should aim to improve quality of life, alleviate suffering and prevent further complications. In severe cases, transitioning to palliative care may be appropriate when aggressive treatment offers limited benefit (Popenhagen et al. 2023).

**Principles of emergency management:** For optimal care, it is crucial to adhere to basic principles that prioritise limiting discomfort and avoiding further complications. A systematic ABCDDEFGH approach which monitors for *A*irways, *B*reathing, *C*irculation, *D*ehydration, *D*iscomfort, *E*ye complications, *F*eeding, *G*enitourinary and *H*aemoglobin, as outlined in Table 2, is recommended to identify and manage emergencies effectively in these vulnerable patients. This structured methodology is essential for guiding emergency care providers through managing common EB emergencies:

**TABLE 2:** Emergency management of patients with epidermolysis bullosa: The ABCDDEFGH approach

Complication and/or emergency	Differential diagnosis	Symptoms and signs	Management
**Airway and Breathing (AB)** Mellerio et al. (2020), Özkan et al. (2016), Saraf et al. (2013)	Acute airway obstruction ■ Obstructive airway blisters ■ Scaring from previous blisters can lead to stenosis at the level of the trachea or larynx Pneumonia ■ Viral ■ Bacterial infection ■ Aspiration	Hoarseness with episodes of stridor Suprasternal and sternal recession Agitation and restlessness Cyanosis or dusky colour Respiratory distress that can lead to respiratory failure	Gently clear the airway of any secretions. *Only suction if needed*; use a soft, small catheter at low-pressure suction. Administer oxygen via nasal prongs rather than a mask, as it causes less pressure to a smaller surface area of the face. High-flow humidified oxygen should be considered early. Non-invasive ventilation can be considered depending on the severity of facial skin involvement. If stridor is present, give adrenaline nebulisation and consider a dose of dexamethasone 0.3 mg/kg – 0.6 mg/kg oral or intravenous, depending on the patient’s clinical condition. Analgesia and anxiolytics are an essential part of symptom control. If patients present with stridor, they should be kept calm and limit agitation. Exclude infection as a cause for respiratory distress and give antibiotics early. In the event of severe respiratory distress with imminent respiratory failure, call for help and consider the overall prognosis of the patient. Respond according to the previously planned level of intervention, if available. *A palliative care plan should be considered in infants with severe JEB, and symptom control should be done rather than invasive airway management. See Palliative care section Part 1.* For patients with less severe forms of EB, with better outcomes, who present with a potentially reversible airway/breathing emergency, urgent airway intervention might be indicated. It is important always to anticipate a potentially difficult airway and prepare accordingly with readily available equipment. Invasive airway management: ■ Use video laryngoscopy or fibreoptic scope if available for intubation. ■ Always lubricate the tubes well. ■ Nasal endotracheal tubes (ETT) are easier to secure, and nasal might be the preferred route for intubation in the presence of oral blisters. ■ Avoid the use of sticky dressings; rather use tracheostomy tape or soft ties with gauze underneath to secure the ETT. ■ If upper airway obstruction indicates intubation, consider a smaller sized endotracheal tube with a small leak to avoid trauma and subglottic stenosis. For induction: Ketamine or propofol with fentanyl can be used. Paralytic agents should be used with caution in patients with upper airway obstruction. First, ensure bag-mask ventilation is possible and that the larynx is visible before paralytic medication is administered. Avoid propofol in patients with sepsis, shock and hypotension.
**Circulation (C)** Mellerio et al. (2020), Department of Health (2023), Liy-Wong et al. (2023) *Patients with shock or impaired circulation should be managed promptly as they can deteriorate rapidly*	Sepsis: Leading cause of death in patients with JEB Shock ■ Septic shock ■ Hypovolaemic shock secondary to poor feeding, diarrhoea, or sepsis Always consider anaemia as a cause of impaired circulation	Tachycardia Delayed capillary refill time of > 2 seconds Cool peripheries Rapid, weak pulses Temperature instability Hypoxia and desaturation Respiratory distress Lethargy Poor end-organ function: poor urine output, depressed level of consciousness Patients with widespread blisters and indwelling catheters will be at higher risk for sepsis.	Give oxygen therapy if saturation is less than 92% or if clinically shocked. Monitor oxygen saturation, respiratory rate and heart rate. Blood pressure monitoring is challenging in severe EB. Hypotension is a late sign of shock in children, and normal blood pressure for age does not exclude shock. Early intravenous access with blood investigations and blood culture. Give broad-spectrum antibiotics – *See antibiotic guidelines.* Consider intravenous infusion (IVI) of fluids early if in shock; Standard Treatment Guidelines for Paediatrics (STG) recommends giving Ringer’s lactate or 0.9% saline 10 ml/kg over 15 min – 20 min and reassess the response. The fluid bolus should be repeated if shock persists. Consider inotropic support after the fourth fluid bolus. Consider anaemia in patients with impaired perfusion and manage it according to the anaemia guideline (see Figure 1). Severe sepsis with shock and multiorgan failure has a poor prognosis in infants with severe generalised EB, and it is appropriate to consider palliative care in these patients. This should be discussed with the family as soon as possible. Where a palliative care plan is already in place, this should be followed.
**Dehydration (D)** Department of Health (2023)	Sepsis Insensible losses via skin in patients with severe EB Poor feeding	Dehydration can be challenging to identify in EB patients. The following EB patients are at higher risk of dehydration: Poor feeding or vomiting Mouth blisters Sepsis Large body surface involvement History of vomiting or diarrhoea Consider severe dehydration in any patient with a history of diarrhoea, vomiting, or poor feeding PLUS any of the following signs: ■ Lethargy ■ Sunken eyes or fontanelle ■ Reduced skin turgor ■ Dry mucosa	Management of dehydration: Always assess the patient for signs of shock first; if in shock, give a 10 ml/kg bolus of Ringer’s lactate or 0.9% saline. If not shocked, estimate the fluid deficit and replace it over 24 h – 48 h. Patients can have mild, moderate (5%), or severe (10%) dehydration. Fluid deficit can be calculated as weight (kg) X % of dehydration X 10. This is the total fluid that should be used for rehydration over 24 h – 48 h. Patients with mild or moderate dehydration of 5% or less can be rehydrated via a nasogastric tube (NGT). An oral rehydration solution can be given via NGT. Patients who were shocked on arrival or severely dehydrated (10%) or those who do not tolerate NGT rehydration should receive IVI fluids for rehydration. The current recommendation from STG for rehydration is Ringer’s lactate OR 0.9% saline with 5% dextrose. Reassess the patient frequently. Stop IVI fluids once tolerating oral feeds and fluids. Check electrolytes and renal function. In patients with hypernatraemic dehydration, fluids should be replaced over 48 h. Intravenous fluids should be used more cautiously in patients with severe malnutrition and anaemia. Rehydration fluids should be adjusted according to the severity of dehydration and ongoing fluid losses (diarrhoea, vomiting and skin loss). Continue to give feeds orally or via NGT if tolerated.
**Discomfort and pain (D)** Goldschneider et al. (2014)	New blisters Infection Corneal erosion Dysphagia Urinary retention	Uncontrollable crying Tachycardia and high blood pressure Tachypnoea Restlessness	Pain and discomfort might be the only reason for a child with EB to present to the ED. Always have a comprehensive approach to EB infants that present in pain. Exclude infection, new blisters, feeding problems and urinary retention. Treat the cause of the discomfort as well as the pain. *See the section on Analgesia in EB, Part 1, consensus statement 13.*
**Eye complications (E)** Mellerio et al. (2020), Fine and Mellerio (2009a)	Local infection Corneal erosion or abrasion can occur in all severe forms of EB	Unable to open eye Excessive tearing Red eyes Pain and discomfort Visual disturbances	Meticulous eye examination; avoid further damage to eyelids and eye Adequate analgesia. Lubrication of the eyes with the use of artificial tears or ointments. Topical antibiotic ointment Eye patches might be required for small children to limit scratches, but adhesive tape should be avoided. *See the section on eye management, consensus statement 19, Table 5.*
**Feeding difficulties (F)** Mellerio et al. (2020), El Hachem et al. (2014)	Mouth blisters with mucosal involvement. Oesophageal blisters or stricture Anaemia, dehydration, pain and new infections can all cause poor feeding	Refuse to feed Excessive drooling Pain and discomfort Crying inconsolable Presence of new or old blisters in mouth	Look for specific causes and exclude any mouth blisters. Give analgesia. Assess for signs of dehydration and manage accordingly. If any new blisters are identified in the mouth, use a finger prick lancet or a hypodermic needle to drain blisters gently. Give NGT feeds if the patient cannot feed. Always look for other causes of poor feeding, such as anaemia, new infection and possible oesophageal involvement if the patient does not improve on the above management. In patients with severe EB with a palliative care plan, the focus should be exclusively on comfort care. In these patients, it might be appropriate to withhold enteral feeds at the end of life.
**Genitourinary (G)** Mellerio et al. (2020), Fine and Mellerio (2009a), Arifi et al. (2011)	Urinary retention Acute kidney injury Urinary tract infection	Discomfort with passing urine Dysuria Dry nappies and anuria Weak urinary stream Abdominal distention with palpable bladder Blisters around the urethral meatus Blood in urine	Management of patients with possible genitourinary complications should include: Give analgesia. In case of a palpable bladder, a urinary catheter should be inserted. (See Basic Principles before inserting catheter.) If unable to pass a urinary catheter, consider ultrasound and insert a suprapubic catheter if an enlarged bladder is confirmed. Check renal function and electrolytes. A urine dipstick should be done, and urine should be sent for culture. Exclude infection and give antibiotics early if a urinary tract infection is suspected. Assess for signs of dehydration, monitor urine output and rehydrate appropriately. The patient should be referred for cystoscopy and further management if urinary obstruction is confirmed. Treat constipation if present.
**Haemoglobin (H)** Liy-Wong et al. (2023)	Multifactorial Anaemia ■ Chronic inflammation ■ Iron deficiency anaemia Sepsis Cardiac failure	Symptomatic anaemia: Weakness and lethargy Tachycardic Weakness Dyspnoea on exertion Cardiac failure Poor feeding Respiratory distress	Hb > 6 g/dL and not symptomatic: Do a full blood count, iron studies and consider iron supplementation once acute infection is excluded. Hb < 6g/dL or symptomatic anaemia: consider transfusion. Packed Red Cells 10 mL/kg – 15 mL/kg over 4 h – 6 h. *See Figure 1*

Note: Please see full reference list of this article: Chateau, A.V., Blackbeard, D., Hlela, C., Wege, M., Armour, L.A., Naicker, T. et al., 2025, ‘Consensus statements for the biopsychosocial care of patients with epidermolysis bullosa South Africa: Part 2’, *Health SA Gesondheid* 30(0), a2964. https://doi.org/10.4102/hsag.v30i0.2964 for more information.

**A non-aggressive approach:** Emergency treatment for EB should emphasise a non-aggressive and minimally invasive approach to balance effective care and minimise pain and complications. Table 2 provides detailed guidelines on the basic management principles applicable to all EB patients in emergency settings.

**The ABCDDEFGH framework:** A structured ABCDDEFGH approach helps address the most common medical emergencies in EB patients, ensuring systematic and efficient care for critically ill children with this rare condition. Table 2 further elaborates on this method and its application in emergency scenarios.

**The ABCDDEFGH approach to the management of epidermolysis bullosa patients in the emergency department:** It is vital to have a structured approach to critically ill children with rare diseases. The ABCDDEFGH approach will help identify and manage the most common medical emergencies in patients with EB.

Table 2 is depicts a detailed approach to the most common emergencies in EB.

Online Appendix 1, Table 1-A1 outlines the targeted antibiotic therapy regarding community- and hospital-acquired infections in patients with EB.

### Nutritional care for patients with epidermolysis bullosa

#### Consensus statement 9: Optimising nutritional status for growth, development and wound healing

Nutrition plays a vital role in a child’s growth, development and wound healing, especially during the critical early years. Any disruptions to adequate nutritional intake can lead to significant failure to thrive (Manjunath et al. 2021).

Patients with EB face unique challenges, including increased nutritional demands because of recurrent infections, chronic inflammation and systemic involvement. Malnutrition can impair wound healing, growth and puberty in these patients (Manjunath et al. 2021).

Aims of nutritional support in EB: The goals of nutritional support as outlined by Salera et al. (2020) include improving nutritional status, easing the burden of oral feeding and addressing dietary deficiencies. This approach promotes growth, bowel function, pubertal development, wound healing and immune system health (Salera et al. 2020).

**Dietary needs of patients with epidermolysis bullosa:** Patients with EB require a higher caloric and protein intake than healthy children. Energy needs are estimated at 100% to 150% of the average requirement for their age (Haynes 2010), while the protein requirement ranges from 115% to 200% (Haynes 2010). Increased protein requirement must be paired with adequate energy to support anabolic processes (Haynes 2010). Micronutrients such as zinc, iron, folate, vitamins A, C, D and B-complex are crucial for growth, wound healing and immune function. Deficiencies in selenium and carnitine may increase the risk of cardiomyopathy (Salera et al. 2020).

**Feeding requirements and supplementation across ages:** For newborns and infants with mild EB, breast milk alone may suffice (El Hachem et al. 2014; Haynes 2010). However, for severe cases with significant energy loss, breast milk and formula may need fortification with commercial feeds to boost energy density while minimising osmotic load to avoid diarrhoea (Haynes 2010). Table 3 provides detailed guidelines on age-specific nutritional supplementation (Salera et al. 2020). Weaning foods can be introduced similarly to healthy children, but avoid hard, abrasive food textures. Foods must be energy-dense and low in bulk to meet increased nutritional needs (Haynes 2010).

**TABLE 3:** Nutritional supplementation at different ages for epidermolysis bullosa patients with higher nutritional requirements.

Age	Feed of choice	Supplement feed of choice with
Newborns/infants	Breast milk	Breast milk fortifier, Maltodextrin powder + lipid powder
	Infant formula	Maltodextrin powder + lipid powder/oil
	Preterm formula	
Children 1–6 years	Eating meals orally (not constipated)	Complete powder nutritional supplements or Ready-to-drink sip feeds (1–1.5 kcal/mL) without fibre
	Eating meals orally (constipated)	Complete powder nutritional supplements with fibre or Ready-to-drink sip feeds (1 kcal/mL – 1.5 kcal/mL) with fibre
	Enteral feeds	Ready-to-feed formulation with 1 kcal/ml according to age with or without fibre, depending on whether the patient is constipated
Children > 6 years	Eating meals orally (not constipated	Complete powder nutritional supplements or Ready-to-drink sip feeds (1.5 kcal/mL – 2.4 kcal/mL) without fibre.
	Eating meals orally (constipated)	Complete powder nutritional supplements with fibre or Ready-to-drink sip feeds (1.5 kcal/mL – 2.4 kcal/mL) with fibre.
	Enteral feeds	Ready to feed formulation with 1.5 kcal/mL – 2.4 kcal/mL according to age with or without fibre, depending on if the patient is constipated or not.

*Source*: Adapted from Salera, S., Tadini, G., Rossetti, D., Grassi, F.S., Marchisio, P., Agostoni, C. et al., 2020, ‘A nutrition-based approach to epidermolysis bullosa: Causes, assessments, requirements and management’, *Clinical Nutrition* 39(2), 343–352. https://doi.org/10.1016/j.clnu.2019.02.023

As the EB disease progresses, achieving satisfactory nutritional intake becomes progressively more difficult for older children. Complications in the oral cavity and oesophagus lengthen meal times and reduce the consistency of food that can be eaten. Natural foods that have been pureed are generally low in energy and nutrients unless consumed in large volumes (Haynes 2010).

For children with small appetites, liquids should be fortified with energy foods (e.g., oil, cheese, butter, peanut butter, mayonnaise, etc.) to increase nutrient density without increasing volumes (Haynes 2008).

Sweet foods and sugary drinks should only be consumed during meals, if necessary, to prevent tooth decay, while savoury snacks are preferred for slow eaters with EB (Haynes 2008).

By tailoring nutritional strategies to the age and severity of EB, healthcare providers can help optimise growth, development and overall quality of life for these patients.

#### Consensus statement 10: Enteral feeding in patients with severe epidermolysis bullosa

For patients with severe EB, who cannot maintain optimal nutritional status because of severe oral lesions or oesophageal strictures, enteral feeding may become essential. Options in this context include nasogastric feeding or gastrostomy (Salera et al. 2020), each requiring special precautions to prevent further complications:

- Nasogastric feeding (Salera et al. 2020): Use a thin gauge tube to minimise damage to the nasal and oesophageal mucosa. Avoid long-term placement and secure the tube using strapping to avoid irritation and friction.
- Gastrostomy (Salera et al. 2020): Maintain meticulous care of the skin at the gastrostomy site to prevent irritation and infection.

**Special considerations**

- For breastfeeding infants: apply petroleum jelly or white soft paraffin to the mother’s nipple and the infant’s lips to reduce friction (El Hachem et al. 2014).
- Soften teats with warm boiled water and enlarge the hole to facilitate easier sucking (El Hachem et al. 2014).
- Use special needs feeders to reduce sucking effort and minimise gum trauma, caused by the bottle collar (Haynes 2010).
- Oesophageal dilation can help improve dietary intake for those with dysphagia (Manjunath et al. 2021).
- Gradually increase the volume and energy content of formulas if growth remains unsatisfactory.

Encourage oral feeding and participation in family meals whenever possible to promote normal eating habits and social interaction.

#### Consensus statement 11: Preventing and managing constipation in patients with epidermolysis bullosa

Constipation in patients with EB can significantly impact the quality of life. Common causes for constipation include low fibre intake, reduced fluid consumption, medication side effects, perianal pain and fear of defaecation in unfamiliar environments.

**Role of fibre in diet:** Fibre is essential in preventing constipation with a recommended daily intake of (age in years) + 5 g/day – 10 g/day (Haynes 2010). However, meeting this requirement can be challenging in patients with extensive oral lesions or dysphagia.

**Effective treatment strategies include** stool softeners, gradual increase in dietary fibre, increased water intake, treatment of perianal lesions to reduce pain and psychological support to address fears related to defaecation. Enemas and suppositories should be avoided in patients with perianal lesions to prevent further discomfort or injury (Hubbard et al. 2020).

#### Consensus statement 12: Clinical and investigative monitoring of macro and micronutrients in epidermolysis bullosa patients

**Clinical monitoring:**

- **Diet history:** assess dietary intake and identify factors affecting nutrition (e.g., constipation, oesophageal strictures, dental issues, gastroesophageal reflux and anal fissures (Sklar & Haynes 2014).
- **Anthropometric measurements:** regularly measure weight, height or length, head circumference and mid-upper arm circumference every 3 to 6 months (Sklar & Haynes 2014). In patients with contractures who cannot stand, use segmental measurements instead of relying on body mass index (BMI), which may not be an accurate tool in EB (Salera et al. 2020).
- **Nutritional Risks:** patients with severe EB are prone to osteopenia and osteoporosis requiring vitamin D and calcium supplementation (Martinez & Mellerio 2010):
  ■ Aim for a haemoglobin level >10 g/dL to reduce anaemia-related complications (Liy-Wong et al. 2023).
  ■ Monitor for hypoalbuminaemia < 30 g/L, which is indicative of nutritional deficiency and poor wound healing (Pope et al. 2012).

**Investigative monitoring (Salera et al. 2020)**

- **Every 6 to 12 months:** Full blood count, electrolytes, liver function test (albumin), calcium, phosphate, magnesium, zinc, iron and vitamin D.

Selenium (in a first-world setting).

- **Annually:**
  ■ Vitamin B1, B12 and folate.
  ■ Carnitine (in a first-world setting).
  ■ Radiographs from age 5 years to monitor for osteopenia (Martinez & Mellerio 2010).

- **Every 1–2 years:** Vitamin E (in a first-world setting).
- **DEXA scan:** conduct an annual DEXA scan to monitor bone mineral density in patients with a bone mineral mass below -2 standard deviation (Salera et al. 2020).

Regular clinical and investigative assessments help to optimise nutritional and overall health outcomes in patients with EB.

### Obstetric care of the pregnant patient with epidermolysis bullosa

#### Consensus statement 13: Diligent antenatal care of women with epidermolysis bullosa is essential to prevent trauma and complications

**Practical aspects in the management of epidermolysis bullosa in pregnancy:** Women with EB face unique challenges during pregnancy, labour and delivery because of the fragility of their skin and the increased risk for complications. This may be because of nutritional deficiencies as well as complications that can arise from changes in skin and mucous membranes resulting in an increased risk for injury during examination in labour and anaesthesia for a caesarean delivery (Araújo et al. 2017; Baloch et al. 2008). Women with EB require specialised care during pregnancy, labour and delivery because of the fragility of their skin, mucosal changes and associated complications. Comprehensive antenatal management minimises trauma and optimises outcomes for both mother and baby. Five key components of antenatal care for women with EB include the following:

- Management of dietary deficiencies:
  ■ Address malnutrition in women with severe EB through dietary interventions improving BMI and correcting micronutrient deficiencies.
  ■ Iron and folate supplementation are essential to treat anaemia (Baloch et al. 2008).
  ■ Laxatives may be needed for constipation.
  ■ Collaboration with a dietician is critical.

- Oral health: Regular dental check-ups are necessary to prevent and manage gingival disease and oral ulcerations, which are common during pregnancy (Ressler-Maerlender et al. 2005).
- Genetic testing: Genetic testing should be made available to all women during pregnancy for informed family planning, Part 1 of the consensus statements.
- Medication review: Assess and adjust medications to safer alternatives at the minimal effective doses to avoid teratogenic risks, especially for conditions such as nausea and vomiting, and gastro-oesophageal reflux in pregnancy (Pillay 2006).
- Antenatal visits: Standard antenatal visits should be adapted to include an early multi-disciplinary team involving maternal-foetal specialists, obstetricians, gynaecologists, dermatologists, dieticians, psychologists, anaesthetists and neonatologists. Develop a thorough antenatal and birth plan in collaboration with the patient (Intong et al. 2017).

Considerations during antenatal visits (Greenblatt et al. 2022):

- Monitor lesions over the distended abdomen, as they may worsen during pregnancy.
- Avoid excessive pressure with the blood pressure cuff and ensure minimal shearing forces when using a tourniquet.
- Use a generous amount of lubricant on the ultrasound probe and gloves and during symphysis-fundal height measurements. Limit vaginal examinations unless essential and always use ample lubrication when required.

By adopting these strategies, antenatal care for women with EB can minimise risks and ensure better maternal and neonatal outcomes.

#### Consensus statement 14: vigilant intrapartum care to reduce the risk of injury in women with epidermolysis bullosa

Women with EB face unique challenges during labour and delivery. Comprehensive planning and a multidisciplinary approach are essential to minimise injury and ensure a safe delivery.

Essential steps in intrapartum care (Greenblatt et al. 2022; Shah et al. 2019):

**General Precautions:**

- Exercising caution during cardiotocograph monitoring.
- Limit vaginal examinations and use a generous amount of lubrication when necessary.
- Avoid instrumental delivery.
- Use non-adhesive dressings to secure the intravenous line and avoid areas of active lesions for venepuncture.
- Avoid unnecessary urinary catherisation.

**Patient handling:**

- Avoid rolling or sliding devices and encourage auto-positioning during transfers.
- Pad the bed and stirrups to reduce pressure.

**Pain management** (Goldschneider et al. 2014; Greenblatt et al. 2022):

- Entonox: lubricate the mask to prevent blistering.
- Epidural or spinal: use non-adhesive strapping for the epidural catheter and handle the site with care.
- General anaesthesia: evaluate for microstomia, oesophageal strictures and oral involvement. Lubricate the lips, avoid fully inflating the cuff of a laryngeal mask airway and be cautious during suctioning.
- Non-pharmacological methods: consider guided breathing techniques and hydrotherapy.

**Mode of delivery:** The choice between vaginal and caesarean section delivery often depends on the psychological concerns and fear of injury or pain (Intong et al. 2017). Foetal outcomes are not affected by the mode of delivery.

**Vaginal delivery:** Safe unless contraindicated by vaginal stenosis, extensive blistering, breech presentation position or other obstetric factors (Greenblatt et al. 2022). Vaginal delivery does not increase the risk of subsequent vaginal scarring or stenosis, even in recessive dystrophic EB (Greenblatt et al. 2022; Intong et al. 2017). External cephalic version for a breech presentation is contraindicated to prevent skin shearing (Greenblatt et al. 2022). Episiotomy should follow standard obstetric indications; healing of tears and episiotomies is typically satisfactory (Greenblatt et al. 2022).

#### Caesarean section delivery in epidermolysis bullosa

Early multidisciplinary consultation, including anaesthesia planning, is essential for caesarean section delivery in EB.

Anaesthesia considerations include:

- Neuraxial anaesthesia is preferred, avoiding excessive disinfectant solution (Greenblatt et al. 2022; Shah et al. 2019).
- Minimise local anaesthesia to prevent bullous formation (Greenblatt et al., 2022; Shah et al. 2019).
- General anaesthesia is typically avoided to reduce the risk of oral and oesophageal damage.

Surgical considerations: (Shah et al. 2019):

- Use bipolar diathermy to avoid adhesive electrocautery pads.
- Avoid adhesive drapes.
- Make a slightly longer incision to minimise trauma during foetal delivery.
- Use subcuticular sutures to minimise skin injury.
- Avoid vigorous stimulation of the neonate.

Careful adherence to these guidelines ensures a safer intrapartum delivery for women with EB and their infants.

#### Consensus statement 15: Postpartum care and discharge planning

A comprehensive postpartum care plan is essential for mothers with EB to ensure recovery, address maternal and neonatal needs and prevent complications.

**Six key postpartum care recommendations:**

- General obstetric guidelines:
  ■ Follow standard obstetric protocols for managing episiotomy and caesarean section wounds (Greenblatt et al. 2022).
- Avoid adhesive dressings on surgical sites to prevent trauma (Greenblatt et al. 2022; Intong et al. 2017)
  ■ Skin-to-skin contact:
  ■ Encourage mother-baby bonding through skin-skin contact.
- Venous thromboembolism prevention:
  ■ Venous thromboembolism is not increased in EB; however, for patients with limited mobility, low molecular weight heparin may be administered. Avoid compression stockings to prevent skin injury.
- Feeding support:
  ■ Counsel patients on feeding options. Breastfeeding is encouraged, with the use of nipple shields or petroleum jelly to protect the nipples. Mixed feeding may be necessary for mothers with debilitating disease (Shah et al. 2019).
- Contraceptive education: discuss and encourage contraceptive use to promote planned pregnancies.
- Comprehensive discharge planning: develop a personalised postpartum care plan involving a psychologist, community clinics and family support to facilitate a smooth transition into motherhood. This multidisciplinary approach ensures maternal well-being and optimal neonatal care.

By addressing these areas, postpartum care can effectively support the physical and emotional health of mothers with EB.

### Sexual and reproductive health and epidermolysis bullosa

#### Consensus statement 16: Health education on sexual health and monitoring for complications

Healthcare practitioners should educate patients on safe sex practices, contraception and sexually transmitted infections, providing guidance and support as they explore safe sexual activities and masturbation (King et al. 2021). The HCP should also monitor for complications such as meatal stenosis, genital blistering, scarring and oral involvement. Lubrication should be recommended to minimise the risk of blister formation (King et al. 2021).

#### Consensus statement 17: Advice for teenagers on menstruation and use of sanitary products

Teenagers with EB may experience premenstrual skin flares or deterioration, as reported by patients. Some choose to use contraceptives to suppress monthly menstrual cycles and alleviate associated symptoms.

**Sanitary towels:** Ill-fitting sanitary towels can cause chafing and blister formation. To prevent discomfort, patients are advised to apply petroleum jelly at the edges of the towel to reduce friction and ensure a proper fit. Frequent changing is essential to avoid contact with damp towels, which can irritate the skin.

**Tampons:** Tampons are preferred option for some patients to avoid issues related to sanitary towels. However, they may not be suitable for individuals with severe disease resulting in vaginal stenosis.

By addressing these considerations, healthcare providers can help patients navigate menstruation with minimum discomfort and skin complications.

#### Consensus statement 18: Medical circumcision is not contraindicated in males with epidermolysis bullosa

Medical circumcision is considered safe for males with EB and may prevent urological complications later in life if performed during infancy (Fine et al. 2004b). Care should be taken to avoid clamps and to protect the surrounding healthy skin during the procedure (Jesus et al. 2014).

### Other multidisciplinary teams involved in the management of patients with epidermolysis bullosa: Eye care, occupational therapy, physiotherapy, orthopaedic management, oral health and footcare

#### Consensus statement 19: Vigilant monitoring for eye symptoms in epidermolysis bullosa is essential

Ocular complications which can lead to blindness are most common and severe in RDEB, JEB, Kindler syndrome and severe EBS; although, all EB subtypes may involve the eyes (Bachir et al. 2022; Figueira et al. 2010; Fine et al. 2004a). A baseline ophthalmology assessment should be conducted immediately after EB diagnosis, with follow-ups determined by severity and type of pathology (Bachir et al. 2022).

Eye pathology severity in EB often correlates with the extent of skin involvement (Bachir et al. 2022) and typically affects superficial structures such as the cornea, conjunctiva and eyelids. Key ophthalmic evaluations include direct inspection of these structures and fluorescein staining at each visit. Table 4 outlines common ocular signs, their associated conditions and treatments.

**TABLE 4:** Common ocular findings in epidermolysis bullosa.

Structure	Clinical finding	Most likely disorder	Treatment summary
Eyelids	Lashes touching the cornea	Entropion or trichiasis	Epilation; surgical correction
	Lid turned outward; watery eye	Ectropion	Surgical correction
	Lids not closing properly + corneal staining	Exposure keratopathy	Lubrication; prolonged patching; surgical correction
	Delayed fluorescein drainage	Nasolacrimal duct obstruction	Probe and syringe; surgical correction
	Crustiness, thickened lids, lid margin telangiectasia	Meibomian gland dysfunction	Scrub lid margins twice daily (ongoing)†; topical antibiotic/steroid and lubricant
Conjunctiva	Fluorescein staining	At risk of symblepharon	Prevention: symblepharon ring‡; glass rodding§
	Lids stuck to each other, or to the globe	Ankyloblepharon or symblepharon	Early: break adhesions with a smooth glass rod
			Late: surgical release and grafts
Cornea	Fluorescein staining on otherwise clear cornea	Corneal erosion	Topical antibiotics; lubrication; prolonged patching
	Fluorescein staining with corneal infiltrate	Corneal ulcer	Intensive topical antibiotics (hourly)¶
	White areas with no fluorescein staining	Corneal scar or opacity	Conservative or surgical management
	Blood vessels growing onto the cornea	Pannus or limbal stem cell deficiency	Lubrication; surgical correction††
	Corneal blisters, often painful	Epithelial bullae	Lubrication; topical antibiotic/steroid; surgical‡‡
	Occasional acute pain, but no corneal findings	Recurrent corneal erosions (epithelium, adherent to the lid, rips on waking)	Lubrication; bandage contact lens; alcohol debridement; stromal micro-puncture

Note: Examples of treatments available in the South African public health sector;

¶ , Antibiotics: G. Ofloxacin; Occ. Chloramphenicol; Occ. Fusithalmic; G. Tobrex; Occ. Tobrex;

†† , Lubricants: G. Tears Naturalle; Occ. Duratears; various antibiotic ointments;

‡‡ , Antibiotic/steroid: G. Spersadex Comp; G. Maxitrol; Occ. Maxitrol; Occ. Tobradex.

† , Lid scrubs: Use diluted baby shampoo (1:10) on a cotton bud or soft cloth to scrub the lid margins twice a day;

‡ , Symblepharon ring: Moulded acrylic barrier ring placed in the fornices to prevent conjunctival adhesions;

§ , Glass rodding: Use the tip of an unopened adrenalin ampule to sweep the fornices daily to break early adhesions.

#### Consensus statement 20: The role of occupational therapy in supporting patients with epidermolysis bullosa

Occupational therapy intervention is vital for individuals with EB to address disabilities such as skin integrity changes, contractures, syndactyly, pain and motor deficits. These challenges can limit independence and hinder participation in meaningful daily activities (Chan et al. 2019).

Therapists help patients regain functional independence and improve participation in home, school, work and community roles by addressing physical, cognitive and sensory-perceptual needs. They employ remedial rehabilitative strategies, adaptive environments and tasks to reduce disability (Chan et al. 2019).

In SA, occupational therapy adapts to challenges such as poverty, resource limitations and rural healthcare access by using effective cost-effective techniques. This approach promotes engagement in daily activities such as bathing and showering, dressing and grooming (Chan et al. 2019), and sexual activity as outlined in Table 2-A1.

Occupational therapy also focuses on early developmental intervention, fine motor skills and hand function to improve quality of life and maintain independence in activities of daily living, particularly for children with EB. Adaptive equipment and task modifications are crucial tools in this process (Eismann et al. 2014).

#### Consensus statement 21: Preventive strategies and management of orthopaedic complications in Recessive Dystrophic Epidermolysis Bullosa

Orthopaedic surgeons are vital members of the multidisciplinary team managing RDEB. Epidermal fragility in RDEB leads to blistering, ulceration, scarring and deformity in the hands and feet (Box et al. 2022), resulting in pain, functional impairment, reduced mobility and decreased quality of life (Eismann et al. 2014). Osteopenia and osteoporosis and fragility fractures are common because of impaired mobility and poor nutrition. Orthopaedic procedures require careful planning to minimise complications.

**Prevention of contractures and pseudosyndactyly**

- Wrap each digit individually with soft silicone foam or paraffin-impregnated gauze in a resource-limited setting to prevent early digit fusion (Denyer, Pillay & Clapham 2017; El Hachem et al. 2014).
- Ensure the thumb is extended and separated from other digits (El Hachem et al. 2014).

**Hand deformities in recessive dystrophic epidermolysis bullosa:** Progressive scarring causes thumb adduction, finger flexion and wrist contractures leading to ‘mitten hand’ in 98% of children with RDEB by the age of 20 years. Multidisciplinary care, hand therapy, splinting and surgery can delay progression (Bernardis & Box 2010, Box et al. 2022).

Surgery aims to restore function, independent finger motion and hand aesthetics (Bernardis & Box 2010). Early intervention provides better outcomes but can still improve function later (Box et al. 2022).

Surgical planning involves decisions on staging, bilateral procedures and soft tissue coverage (Box et al. 2022). Full-thickness skin grafts (FTSG) are preferred in children, while split-thickness skin grafts are common in adults, despite a higher recurrence rate (Bernardis & Box 2010).

**Foot deformities in recessive dystrophic epidermolysis bullosa:** Foot deformities, including toe extension contractures, ankle equinus and cavus deformity, cause pain and decreased mobility. Surgery is indicated before joint degeneration to optimise mobility (Sternick et al. 2016). Pseudosyndactyly release of the toes is rarely performed because of limited functional benefit and high recurrence rate.

**Surgical considerations**

Correct nutritional deficits and ensure haemoglobin levels exceed 10 g/dL (Box et al. 2022).

Exclude β-haemolytic streptococcal skin infection with preoperative skin swab cultures, as it is a contraindication (Box et al. 2022).

Surgical intervention remains critical to improving mobility, hand function and overall quality of life, despite the risk of recurrence (Box et al. 2022).

#### Consensus statement 22: Physiotherapy for patients with epidermolysis bullosa: enhancing mobility, preventing complications and promoting inclusivity

Physiotherapy plays a critical role in managing EB by preventing complications such as contractures and deformities, promoting mobility, maintaining autonomy and enabling inclusivity (El Hachem et al. 2014).

Goals of physiotherapy (Mullett & Atherton 1990; Weisman et al. 2021):

- Developmental milestones: support patients in achieving motor milestones
  ■ Blister formation:
    ◦ Handle neonates gently by the neck and buttocks.
    ◦ Place infant on non-frictional surfaces.
    ◦ Use protective dressings on the hands and knees during crawling and feet during walking.
  ■ Prone positioning – prevent soft tissue shortening and adherence (Mullett & Atherton 1990).
  ■ Motor development – monitor progress and provide development programmes.

- Exercise and functional mobility programmes:
  ■ Prevent or manage contractures through gentle exercises or stretching.
  ■ Strengthen and balance muscles.
  ■ Teach families how to perform exercises at home.
  ■ Provide wrapping techniques that facilitate movement.

- Promote safe weight-bearing:
  ■ Advise on footwear and knee padding.
  ■ Assess joint range, muscle power, balance and gait.
  ■ Assist with weight-bearing, standing frames and strengthening activities.
  ■ Optimising bone and cardiovascular health through weight-bearing exercises.

- Enhance functional mobility:
  ■ Encourage ambulation and endurance activities.
  ■ Focus on foot exercises.
  ■ Gait correction and mobility aids and prostheses.
  ■ Optimise cardiorespiratory health using breathing exercises, percussion and postural drainage to prevent infections (Mullett & Atherton 1990).

**Hand therapy:** Assess hand contractures every 6 to 12 months using tools such as the Assessment of Hand Contractures in EB (Box et al. 2022). Surgery may be required to release contractures (discussed precedingly). Manage pseudosyndactyly with stretches, exercises and splinting (Mullett & Atherton 1990). Splints can increase the range of movement and delay contractures, typically worn at night or for a short daytime period if night-time is not tolerated. Care is needed to prevent blistering from splints (Box et al. 2022).

Physiotherapy fosters independence and improves quality of life by addressing physical challenges while empowering patients and their families.

#### Consensus statement 23: Good oral health, trauma prevention and monitoring for oral complications in epidermolysis bullosa

Dental health is a critical aspect of care for EB patients, yet many dental practitioners may be unfamiliar with the condition (Krämer et al. 2020). Resource-limited settings such as SA face challenges in providing specialised dental care for patients with complex needs.

**Importance of dental care:** Early referral to a dentist is essential for preventing complications and promoting good oral hygiene. Proper dental care helps reduce pain, ensures the ability to eat and maintain nutrition, supports speech and phonation, improves cosmesis and enhances quality of life.

Oral complications in EB can lead to pain, difficulty in chewing, reduced oral intake, speech challenges and psychological effects such as low confidence and poor self-image (Feijoo et al. 2011). Dental caries often results from poor hygiene because of pain, reliance on high-caloric foods, frequent meals, delayed food clearance and medications with high sugar content. Collaboration with a dietician is crucial (Krämer et al. 2020).

The clinical presentation and complications vary as per subtype of EB. Oral complications were discussed in Part 1. Table 5 discusses the dental medical management of EB patients (Has et al. 2021; Krämer et al. 2020).

**TABLE 5:** Dental management of patients with epidermolysis bullosa.

Management	Management strategies
History Exam	Frequency of brushing. Diet. Difficulties and complications.
Exam	Caries, enamel, gum care, palate, tongue, microstomia, ankyloglossia. Bleeding, erosions, blisters, atrophy.
Preparation and preventative strategies	Correct positioning on the dentist’s chair. Cushioning with non-adhesive foam. Non-adhesive dressings on the lips and chin to prevent shearing force. Use petroleum jelly or glycerine on the lips and at the back of instruments when examining the mouth. Suction should be placed on a tooth, not on the mucosa, as this can induce trauma. Avoid high-pressure suctioning. Small cotton rolls lubricated with water-soluble lubricants.
Treatment	Gently clean using a small toothbrush. An electric toothbrush may be advantageous to prevent hand movement. Use fluorinated toothpaste and avoid irritants. Xylitol chewing gum has been used as a preventative strategy for patients at substantial risk of dental caries. Rinse after each meal. Floss daily. Sucralfate reduces pain and blister formation. Topical anaesthetics such as 1% lidocaine or 2% xylocaine gel. Seal fissures with resin or glass ionomer. Microstomia can limit speech, impede eating and make dental procedures and intubation very difficult. ■ Use paediatric-size instruments during procedures. ■ Daily exercises with mechanical devices that allow an incremental increase in mouth aperture. ■ Surgical release of contractures. Minimally invasive dental techniques are advocated for all forms of EB. Gentle endodontic, periodontal and orthodontic care.
Monitoring	3 to 6-month appointments monitoring for: ■ Caries. ■ Non-healing ulcers, red or white lesions that may represent squamous cell carcinoma, especially in patients with RDEB and Kindler syndrome.

Note: The references are mentioned alongside the comment. Refer the reader to guidelines regarding anaesthesia and implants: https://www.debra-international.org/eb-health-care-cpgs. Parent information regarding oral care; https://www.debra.org/more-eb-guides/oral-health-care.

**Management:** Planning appointments: dental visits should be well organised, with staff trained to prevent trauma and address the anxiety of patients and their families.

Specialised care: restorative, endodontic, orthodontic and periodontal treatments are not contraindicated in EB patients and require careful handling (Feijoo et al. 2011; Krämer et al. 2020).

Good oral health practices and vigilant monitoring help mitigate the significant challenges associated with oral complications in EB, supporting overall well-being and quality of life.

#### Consensus statement 24: Foot care in epidermolysis bullosa – Prevention and management

Careful foot care is essential for all EB subtypes to prevent new blisters, manage existing ones and address complications. Up to 90% of EB patients experience podiatric issues, including dystrophic nails, hyperkeratosis, blisters, foot deformities and flat feet (Khan et al. 2020). These manifestations can be painful and significantly impact mobility and quality of life. A podiatrist plays a vital role in the multidisciplinary team providing targeted care. Table 6 outlines strategies for blister prevention, management of complications and support for motor skill development to enhance patient outcomes (DEBRA International; Khan 2010; Khan et al. 2020).

**TABLE 6:** Podiatry care for patients with epidermolysis bullosa.

Management	Management strategies
Foot care	Keep feet clean and dry. Soak feet in salt water before cutting nails. Nails must be trimmed straight across. Apply baby oil to the nail to prevent nail thickening. Monitor for the fusion of the toes; use gel pads to separate toes.
**Education to prevent blister formation**	
Footwear	Encourage comfortable shoes with a rounded toe, laces or straps and seamless internal lining to prevent excess movement and friction. Custom orthotics and shock-absorbing insoles. Avoid shoes that will retain heat and precipitate blister formation. Leather shoes are preferred to allow for ventilation. Rub petroleum jelly or oil to make them soft.
Socks	Provides ventilation. Absorbs moisture. Reduces friction. Silver-lined. Double layer of socks – prevents friction.
Decrease moisture	Apply corn flour in socks to prevent excessive moisture.
Manage blisters	Lance blisters and allow to drain. Dressings depend on the type of wound (see wound care section).
**Complications**	
Dystrophic nails	Trim toenails straight across. Soak feet in salt water or during a bath. File the nail surface with an emery board thereafter. Apply keratolytics such as urea; the concentration will depend on the patient’s age and the thickness of the nail.
Hyperkeratosis (callus) and corns	Emory board to pare the hard skin. If this fails, then referral to a podiatrist to pare down the lesions with a scalpel. Apply emollients post-paring and non-adherent dressing. Careful not to over-debride/pare, as this could lead to blister formation. Do not use corn plasters; they adhere to the skin and contain acidic material that can cause maceration.
Deformity and syndactyly	Refer to orthopaedic surgeon for reconstruction.
Motor skill development for children	Children can walk barefoot indoors with or without socks. Extra padding over dressings on friction sites when the child starts crawling or walking.

*Source*: Khan, M.T., 2010, ‘Podiatric management in epidermolysis bullosa’, *Dermatologic Clinics* 28(2), 325–333. https://doi.org/10.1016/j.det.2010.02.006; Khan, M.T., O’sullivan, M., Faitli, B., Mellerio, J.E., Fawkes, R., Wood, M. et al., 2020, ‘Foot care in epidermolysis bullosa: Evidence-based guideline’, *British Journal of Dermatology* 182(3), 593–604. https://doi.org/10.1111/bjd.18381; Debra International, 2018, *Foot care in epidermolysis bullosa*, viewed 29 November 2024, from https://www.debra-international.org/foot-care-in-eb

**Global perspective:** While the discussion focuses on SA, these consensus statements might inform guidelines for other resource-constrained settings through future collaboration.

**Future directions**

- Encourage HCPs to partner with DEBRA SA.
- The consensus statements must be readily available to HCPs.
- Continued medical education regarding the care of patients with EB.
- Further research and collaboration with local and international experts.
- Schools should be adapted and cater to children with high needs and disabilities. Children with EB should be integrated into mainstream schools that accommodate their limitations.
- Societies, communities and schools need to be educated about EB to prevent stigma and bullying and foster acceptance of patients with EB.
- Continuity of care in the healthcare sector is vital to ensure effective and comprehensive care of patients, which will decrease anxiety among patients. An effective transition of care is essential when moving between paediatric and adult healthcare services.

## Conclusion

Epidermolysis bullosa is a multisystemic inherited condition that can have a profound effect on the patient and their families and requires a transdisciplinary approach for the comprehensive care of these patients. Part 1 outlined 16 consensus statements for the diagnostic and clinical care of EB, informed by a range of dermatologists and paediatricians. Part 2 developed 24 consensus statements around the biopsychosocial aspects of EB care. Together, Part 1 and Part 2 of the consensus statements provide a contextually unique approach to managing EB in SA with relevance to other resource-limited settings. The consensus statements also represent a preliminary intervention to provide a detailed framework upon which knowledge and experience in the transdisciplinary and holistic management of EB can be further developed and refined. These consensus statements provide a preliminary and practical framework for managing EB that is relevant to the South African cultural and social landscape. An aspiration is that this framework may have practical and contextual relevance for similar resource-limited settings. Ongoing research with advocacy for and inclusion of persons living with EB may lead to the improvement and further development of these consensus recommendations.
